# Associations of bilirubin, albumin, and the ALBI index with gastric cancer risk: a smoking-stratified prospective cohort study in men

**DOI:** 10.1186/s12876-026-05002-1

**Published:** 2026-06-11

**Authors:** Jong Won Shin, Ji-Won Lee, Nguyen Thien Minh, Sun Ha Jee

**Affiliations:** 1 Department of Laboratory Medicine, Asan Medical Center, University of Ulsan College of Medicine, Ulsan, Seoul, Republic of Korea; 2 Department of Epidemiology and Health Promotion, Institute for Health Promotion, School of Public Health, Yonsei University, Seoul, Republic of Korea; 3 Department of Family Medicine, Severance Hospital, Yonsei University College of Medicine, Seoul, 03722 Republic of Korea; 4 Institute for Innovation in Digital Healthcare, Yonsei University, Seoul, 03722 Republic of Korea; 5 Department of Epidemiology, Faculty of Public Health, University of Medicine and Pharmacy, Ho Chi Minh City, Viet Nam; 6 Department of Transdisciplinary Healthcare Sciences, Graduate School of Transdisciplinary Health Science, Yonsei University, Seoul, 03722 Republic of Korea

**Keywords:** Bilirubin, Albumins, Gastric Neoplasms, Oxidative Stress, Smoking

## Abstract

**Background:**

Bilirubin and albumin are endogenous antioxidants, and the ALBI index may reflect systemic oxidative stress. However, their associations with gastric cancer risk, especially by smoking status in healthy populations, remain unclear.

**Methods:**

This study utilized data from the KCPS-II cohort, including a total of 83,371 men participants without cancer at baseline. During a mean follow-up period of 13.5 years, 1,162 cases of gastric cancer were identified. Serum total bilirubin, albumin, and the ALBI index were analyzed according to smoking status. Cox proportional hazards regression models were used to estimate hazard ratios (HRs) and 95% confidence intervals (CIs), with additional analyses conducted using quartiles and trend tests.

**Results:**

A 1 SD increase in total bilirubin was significantly inversely associated with gastric cancer among ever-smokers (HR: 0.80, 95% CI: 0.65–0.98). A similar inverse association was observed among former smokers (HR: 0.73, 95% CI: 0.53–1.00). Albumin showed the strongest inverse association with gastric cancer among ever-smokers (HR: 0.59, 95% CI: 0.45–0.77), with consistent associations observed in both current and former smokers (HR: 0.60, 95% CI: 0.42–0.86; HR: 0.62, 95% CI: 0.41–0.92, respectively). The ALBI index was also significantly inversely associated with gastric cancer among ever-smokers (HR: 0.74, 95% CI: 0.56–0.99). In contrast, none of the three biomarkers showed statistically significant associations with gastric cancer among never smokers.

**Conclusions:**

This study suggests that serum bilirubin, albumin, and the ALBI index may serve as useful antioxidant biomarkers for predicting gastric cancer risk, particularly showing strong inverse associations among smokers.

**Supplementary Information:**

The online version contains supplementary material available at 10.1186/s12876-026-05002-1.

## Background

Gastric cancer remains a major global health burden, ranking among the leading causes of cancer-related mortality worldwide [1, 2]. Notably, the pathogenesis of gastric cancer may fundamentally differ according to smoking status. Smoking-related gastric cancer is primarily driven by the accumulation of reactive oxygen species (ROS) generated by smoking and the resulting oxidative stress, which damages the gastric mucosa and promotes cellular mutations that accelerate carcinogenesis [3–7]. Bilirubin and albumin are potent endogenous antioxidants that may inhibit key steps in this carcinogenic process by neutralizing ROS. Specifically, bilirubin, a product of heme catabolism, possesses strong ROS-scavenging properties and has been shown to suppress oxidative DNA damage, lipid peroxidation, and activation of the NF-κB signaling pathway, thereby exerting anti-inflammatory and anticarcinogenic effects [8, 9]. Albumin contributes to antioxidant and anti-inflammatory defense by binding to transition metal ions (e.g., iron, copper), inhibiting the Fenton reaction, and directly neutralizing free radicals [10]. These antioxidant mechanisms may help alleviate oxidative stress in the gastric mucosa and thereby reduce the risk of gastric cancer development, particularly in smoking-induced environments.

In contrast, gastric cancer in never-smokers is more commonly associated with multifactorial causes such as Helicobacter pylori infection, dietary factors, genetic susceptibility, and chronic inflammation. Chronic H. pylori infection is closely linked to gastric ulcers, atrophic gastritis, and peptic ulcer disease [11, 12] and the World Health Organization classified it as a Group I carcinogen in 1994 due to its strong association with gastric cancer [13]. These differences underscore the need for a stratified approach in understanding gastric cancer risk and developing prevention strategies based on smoking history.

This study utilized data from the Korean Cancer Prevention Study-II (KCPS-II), a large-scale prospective cohort, and performed stratified analyses according to smoking status while adjusting for a range of potential confounders. These included clinical indicators such as body mass index (BMI) and liver enzymes (GOT, GGT), as well as socioeconomic factors including family history of cancer, educational attainment, and household income. Notably, gamma-glutamyl transferase (GGT) has been implicated in recent studies as a biological mediator in H. pylori-associated gastric carcinogenesis, by altering energy metabolism and histone methylation status in gastric epithelial cells [14], thereby supporting its inclusion as an adjustment variable in this analysis.

Previous studies have suggested that higher serum bilirubin levels are associated with a reduced risk of pulmonary function decline, lung cancer, and cardiovascular disease, with these associations being more prominent among smokers [15–19]. The albumin-bilirubin (ALBI) index, originally developed to assess liver function and predict prognosis in hepatocellular carcinoma [20], has recently been applied to various solid tumors [11, 15, 21–25]. While several studies have reported that bilirubin, albumin, and the ALBI index are associated with prognosis in gastric cancer patients, most have relied on cross-sectional designs involving post-diagnosis patient populations. Such designs are vulnerable to confounding by treatment status, cancer stage, and nutritional conditions, limiting the ability to evaluate the independent role of these biomarkers. Importantly, there is a paucity of prospective, population-based studies that examine the associations of bilirubin, albumin, or the ALBI index with gastric cancer risk, particularly among smokers. Therefore, this study aimed to investigate the associations of serum bilirubin, albumin, and the ALBI index with the risk of gastric cancer in men, stratified by smoking status, using data from the KCPS-II cohort. By exploring the potential antioxidant roles of these biomarkers under conditions of smoking-induced oxidative stress, this study seeks to provide novel evidence from a preventive perspective, complementing previous research that has focused primarily on prognosis.

## Methods

### Study population

This study was based on data from the Korean Cancer Prevention Study-II (KCPS-II), a large-scale prospective cohort comprising 153,971 adults aged 20 years or older who underwent health examinations at 18 medical centers across South Korea between 2004 and 2013. All participants provided written informed consent for the use of their health screening and questionnaire data for research purposes [26, 27]. Of the initial 153,971 participants, a total of 20,341 individuals (13.2%) were excluded due to a history of cancer at baseline or missing data on key variables, including smoking status, alcohol consumption, and serum levels of bilirubin and albumin. Missing values were handled through complete case exclusion without imputation in order to minimize bias and maintain the integrity of the dataset (Supplementary Fig. 1). The primary objective of this study was to evaluate the associations between serum levels of bilirubin, albumin, and the albumin-bilirubin (ALBI) index with the risk of gastric cancer according to smoking status. Since smoking-related gastric cancer is more prevalent among men and presents with lower biological heterogeneity, the main analysis focused on male participants. A total of 83,371 men (62.4%) and 50,259 women (37.6%) were included in the sex-stratified analyses. While the primary findings presented in this manuscript are based on men, the results including both men and women are provided in the Supplementary Table 1. Women were not included in the primary statistical models due to their low smoking prevalence and correspondingly lower incidence of smoking-related gastric cancer, which limited statistical interpretability. This study was approved by the Institutional Review Board of Severance Hospital, Yonsei University Health System (approval number: 4–2011–0277) and was conducted in accordance with the Declaration of Helsinki.

### Bilirubin measurement

Bilirubin levels were measured using an automated chemistry analyzer (AU5800; Beckman Coulter, Seoul, Korea). Total bilirubin levels are determined through the reaction of bilirubin with a stabilized diazonium salt, specifically 3,5-dichlorophenyldiazonium tetrafluoroborate (DPD), resulting in the formation of azobilirubin. Caffeine and surfactants are incorporated to accelerate this reaction. The azobilirubin is then measured based on its absorbance at 570/660 nm, with this absorbance being proportional to the bilirubin concentration in the sample. To correct for any endogenous serum interference, a separate serum blank is also measured. The within-run precision of the assay has a coefficient of variation (CV) of less than 3% or a standard deviation (SD) of ≤ 0.07, while the total precision maintains a CV of less than 5% or an SD of ≤ 0.10. The measurement of direct bilirubin employs a modified version of the classical method developed by Van den Bergh and Mueller [28]. In this method, direct (conjugated) bilirubin reacts with DPD in an acidic environment to produce azobilirubin. The serum concentration of direct bilirubin is proportional to the intensity of the azobilirubin color, which is measured at 540/600 nm. The within-run precision for direct bilirubin measurements displays a CV of less than 7% or an SD of ≤ 0.07, while the total precision exhibits a CV of less than 8% or an SD of ≤ 0.21. Indirect bilirubin is not measured, but rather is calculated as the difference between total bilirubin and direct bilirubin.

### Cancer case ascertainment

The occurrence of cancer among study participants was verified annually with nearly 100% accuracy by linking data from the National Cancer Center (NCC) Registry using resident registration numbers [26]. In Korea, under the Cancer Control Act, all hospitals are mandated to report cancer diagnoses to the NCC. Cancer cases were classified according to the 10th revision of the International Classification of Diseases (ICD-10). The average follow-up duration for the cohort was 13.5 years. Specifically, for gastric cancer (ICD-10 code C16), the median follow-up time among men was 13.7 years (IQR: 13.3–14.4) for never-smokers, 14.1 years (IQR: 13.4–14.7) for ex-smokers, and 14.1 years (IQR: 13.4–14.6) for all smokers. A total of 1,162 incident cases of gastric cancer were identified during the follow-up period.

### Statistical analysis

Baseline characteristics of the study population were summarized using descriptive statistics according to smoking status (never smokers, former smokers, current smokers, and ever smokers). Among ever smokers, smoking exposure was additionally quantified using smoking duration (years), smoking intensity (cigarettes per day), and pack-years. Pack-years were calculated as the number of cigarettes smoked per day divided by 20 multiplied by smoking duration in years. Each biomarker, including total bilirubin, albumin, and the ALBI index, was standardized by its standard deviation (SD), and Cox proportional hazards models were used to evaluate the association between a 1-SD increase in each biomarker and the risk of gastric cancer. All models were adjusted for potential confounders, including age, alcohol consumption, body mass index (BMI), glutamate oxaloacetate transaminase (GOT), gamma-glutamyl transferase (GGT), and family history of cancer. In a sensitivity analysis, we additionally adjusted for socioeconomic factors such as income level and educational attainment. Subsequently, each biomarker was categorized into quartiles, with the lowest quartile serving as the reference group. Hazard ratios (HRs) and 95% confidence intervals (CIs) were calculated, and linear trends across quartiles were tested by assigning the median value of each quartile and modeling it as a continuous variable. Quartile cutoff values were defined based on the distribution among male participants as follows: Total bilirubin: ≤ 0.7, 0.71–0.9, 0.91–1.1, and > 1.1 mg/dL, Albumin: ≤ 4.4, 4.5–4.6, 4.7, and > 4.7 g/dL, ALBI index: ≤ –3.2662, –3.2661 to –3.1135, –3.1134 to –2.9686, and > –2.9686. The ALBI index was calculated using the following formula: ALBI = (log₁₀ bilirubin [µmol/L] × 0.66) + (albumin [g/L] × –0.085) [20]. To apply this formula, bilirubin levels were converted from mg/dL to µmol/L (1 mg/dL = 17.1 µmol/L). To minimize the potential for reverse causality due to cancer latency, a sensitivity analysis was performed by excluding gastric cancer cases diagnosed within the first three years of follow-up. All statistical analyses were conducted using SAS version 9.4 (SAS Institute Inc., Cary, NC, USA), and person-years were used as the time scale from baseline to the date of gastric cancer diagnosis, death, or end of follow-up. This study was designed and reported in accordance with the STROBE guidelines.

## Results

### Characteristics of the study population

As shown in Table 1, the study population included 133,596 participants, of whom 83,371 were men. The mean age was 40.9 years (SD: 10.0) overall and 41.6 years (SD: 9.5) among men. The variables analyzed in this study included body mass index (BMI), serum bilirubin (total, indirect, and direct), albumin, platelet count, alcohol consumption, and smoking status. Smoking status was categorized as never-smokers, former smokers, and current smokers, while alcohol consumption was grouped into never-drinkers, former drinkers, and current drinkers. Among ever-smoking men, the mean smoking duration was 16.9 years, the mean smoking intensity was 15 cigarettes per day, and the mean cumulative smoking exposure was 13.7 pack-years.

**Table 1:** Baseline characteristics of Korean cancer prevention study-II participants^*^

Characteristics	Men (*n* = 83,371)	Women (*n* = 50,225)
Age, y	41.6 (9.5)	39.7 (10.7)
Body mass index†	24.4 (2.9)	22.0 (3.0)
Serum bilirubin, mg/dL		
Total	0.95 (0.38)	0.75 (0.30)
Indirect	0.59 (0.26)	0.47 (0.21)
Direct	0.36 (0.14)	0.29 (0.12)
Albumin, g/dL	4.58 (0.25)	4.45 (0.25)
Platelet count, × 10^3^/µL	246.27 (52.72)	257.82 (57.81)
Alcohol drinking, g/d	22.67 (29.45)	5.98 (13.77)
Smoking status, %		
Never	22.7	89.4
Previous	32.7	6.4
Current	44.6	4.2
Any alcohol use, %		
Never	5.8	30.8
Previous	7.9	16.5
Current	86.3	52.7

^*^Data are expressed as mean (SD) unless otherwise indicated. Participants with any of the following features at study entry were excluded: missing data on serum bilirubin level, existing cancer, and missing data on smoking status

^†^Body mass index was calculated as weight in kilograms divided by the square of height in meters

**Fig. 1:**
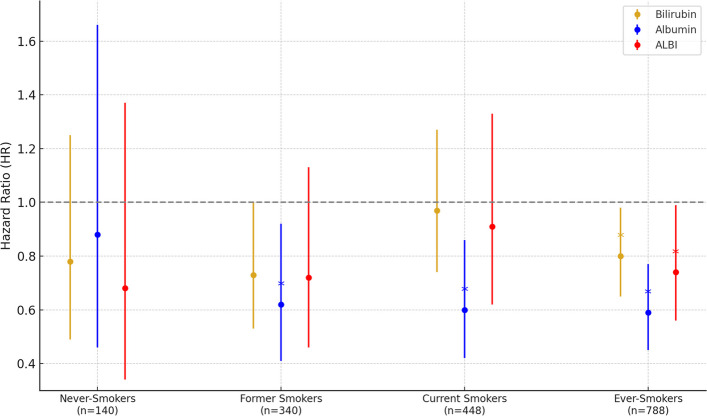
Associations of a 1-SD increase in bilirubin, albumin, and ALBI with gastric cancer risk by smoking status in men

In men, total bilirubin was not significantly associated with gastric cancer risk among never smokers and current smokers. A borderline significant inverse association was observed in former smokers (HR: 0.73, 95% CI: 0.53–1.00, *p* = 0.0512), and a statistically significant inverse association was found in ever smokers (HR: 0.80, 95% CI: 0.65–0.98, *p* = 0.0279). Serum albumin showed no significant association with gastric cancer risk among never smokers. A significant inverse association was observed in former smokers (HR: 0.62, 95% CI: 0.41–0.92, *p* = 0.0181), and a strong inverse association was found in current smokers (HR: 0.60, 95% CI: 0.42–0.86, *p* = 0.0051). Similarly, a strong inverse association was also observed in ever smokers (HR: 0.59, 95% CI: 0.45–0.77, *p* < 0.0001). The ALBI index was not significantly associated with gastric cancer risk among never smokers, former smokers, or current smokers. However, a significant inverse association was observed in ever smokers (HR: 0.74, 95% CI: 0.56–0.99, *p* = 0.0422) (Table 2, Fig. 1).

**Table 2:** Associations of a 1-SD Increase in Bilirubin, Albumin, and ALBI Index with Gastric Cancer Risk According to Smoking Status in Men

Biomarkers	Smoking Status	Cases (Median Follow-Up Time, IQR)	HR (95% CI) §	*p*-value
Bilirubin	Never-Smokers	140 (13.7, 13.3–14.4)	0.78 (0.49–1.25)	0.3013
	Former Smokers	340 (14.1, 13.4–14.7)	0.73 (0.53–1.00)	0.0512
	Current Smokers	448 (14.1, 13.4–14.6)	0.97 (0.74–1.27)	0.8066
	Ever-Smokers*	788 (14.1, 13.4–14.6)	0.80 (0.65–0.98)	0.0279
Albumin	Never-Smokers	140 (13.7, 13.3–14.4)	0.88 (0.46–1.66)	0.6887
	Former Smokers	340 (14.1, 13.4–14.7)	0.62 (0.41–0.92)	0.0181
	Current Smokers	448 (14.1, 13.4–14.6)	0.60 (0.42–0.86)	0.0051
	Ever-Smokers*	788 (14.1, 13.4–14.6)	0.59 (0.45–0.77)	< 0.0001
ALBI	Never-Smokers	140 (13.7, 13.3–14.4)	0.68 (0.34–1.37)	0.2828
	Former Smokers	340 (14.1, 13.4–14.7)	0.72 (0.46–1.13)	0.1571
	Current Smokers	448 (14.1, 13.4–14.6)	0.91 (0.62–1.33)	0.6191
	Ever-Smokers*	788 (14.1, 13.4–14.6)	0.74 (0.56–0.99)	0.0422

*Abbreviations*: *CI* confidence interval, *HR* hazard ratio

^*^Ever-smokers include both current and former smokers

^§^The Cox proportional hazards model was adjusted for age, alcohol use, body mass index, GOT, and GGT, family history of cancer

In the quartile-based analysis, we evaluated the associations of each biomarker with gastric cancer risk stratified by smoking status. For total bilirubin, no significant trends were observed among never, former, or current smokers. However, in ever smokers, a significant inverse linear trend was identified (p for trend = 0.0019), with Q3 (HR: 0.81, 95% CI: 0.67–0.98) and Q4 (HR: 0.75, 95% CI: 0.60–0.92) demonstrating statistically significant inverse associations. For albumin, while no significant trend was found among never smokers, Q4 showed a significant inverse association in both former smokers (HR: 0.70, 95% CI: 0.52–0.94) and current smokers (HR: 0.68, 95% CI: 0.52–0.90, p for trend = 0.0135). Among ever smokers, significant inverse associations were observed in Q3 (HR: 0.81, 95% CI: 0.68–0.97) and Q4 (HR: 0.68, 95% CI: 0.55–0.83), along with a strong linear trend (p for trend < 0.0001). For the ALBI index, no significant trends were noted among never, former, or current smokers. However, in ever smokers, a significant inverse association was observed in Q4 (HR: 0.76, 95% CI: 0.62–0.93), accompanied by a significant linear trend (p for trend = 0.0034) (Table 3).

**Table 3:** Associations of Bilirubin, Albumin, and ALBI Index with Gastric Cancer Risk by Smoking Status in Men: Quartile and Trend Analyses

Biomarkers	Smoking Status	Case	Q1 HR (95% CI) §	Q2 HR (95% CI) §	Q3 HR (95% CI) §	Q4 HR (95% CI) §	*p-*value for Trend
Bilirubin	Never-Smokers	140	1	0.82 (0.51–1.33)	0.73 (0.45–1.17)	0.83 (0.51–1.34)	0.3910
	Former Smokers	340	1	0.92 (0.67–1.26)	0.89 (0.66–1.20)	0.77 (0.55–1.07)	0.1213
	Current Smokers	448	1	1.01 (0.79–1.29)	0.81 (0.63–1.04)	0.83 (0.62–1.10)	0.0641
	Ever-Smokers*	788	1	0.95 (0.78–1.15)	0.81 (0.67–0.98)	0.75 (0.60–0.92)	0.0019
Albumin	Never-Smokers	140	1	1.14 (0.69–1.89)	1.09 (0.69–1.64)	1.02 (0.64–1.62)	0.9409
	Former Smokers	340	1	0.83 (0.61–1.14)	0.77 (0.58–1.01)	0.70 (0.52–0.94)	0.0113
	Current Smokers	448	1	0.80 (0.61–1.06)	0.87 (0.69–1.10)	0.68 (0.52–0.90)	0.0135
	Ever-Smokers*	788	1	0.81 (0.65–0.99)	0.81 (0.68–0.97)	0.68 (0.55–0.83)	0.0001
ALBI	Never-Smokers	140	1	0.98 (0.62–1.55)	0.76 (0.47–1.24)	0.88 (0.55–1.41)	0.4135
	Former Smokers	340	1	0.95 (0.70–1.27)	0.94 (0.70–1.27)	0.79 (0.58–1.08)	0.1620
	Current Smokers	448	1	1.04 (0.83–1.32)	0.83 (0.64–1.08)	0.83 (0.63–1.09)	0.0745
	Ever-Smokers*	788	1	0.97 (0.81–1.17)	0.84 (0.69–1.02)	0.76 (0.62–0.93)	0.0034

*Abbreviations*: *CI* confidence interval, *HR* hazard ratio

^*^Ever-smokers include both current and former smokers

^§^The Cox proportional hazards model was adjusted for age, alcohol use, body mass index, GOT, and GGT, family history of cancer

In the sensitivity analysis stratified by income and education level, albumin remained statistically significant among current smokers (HR: 0.41, 95% CI: 0.24–0.69), ever smokers (HR: 0.51, 95% CI: 0.35–0.75), and all men (HR: 0.58, 95% CI: 0.40–0.83) (Supplementary Table 1).

**Fig. 2:**
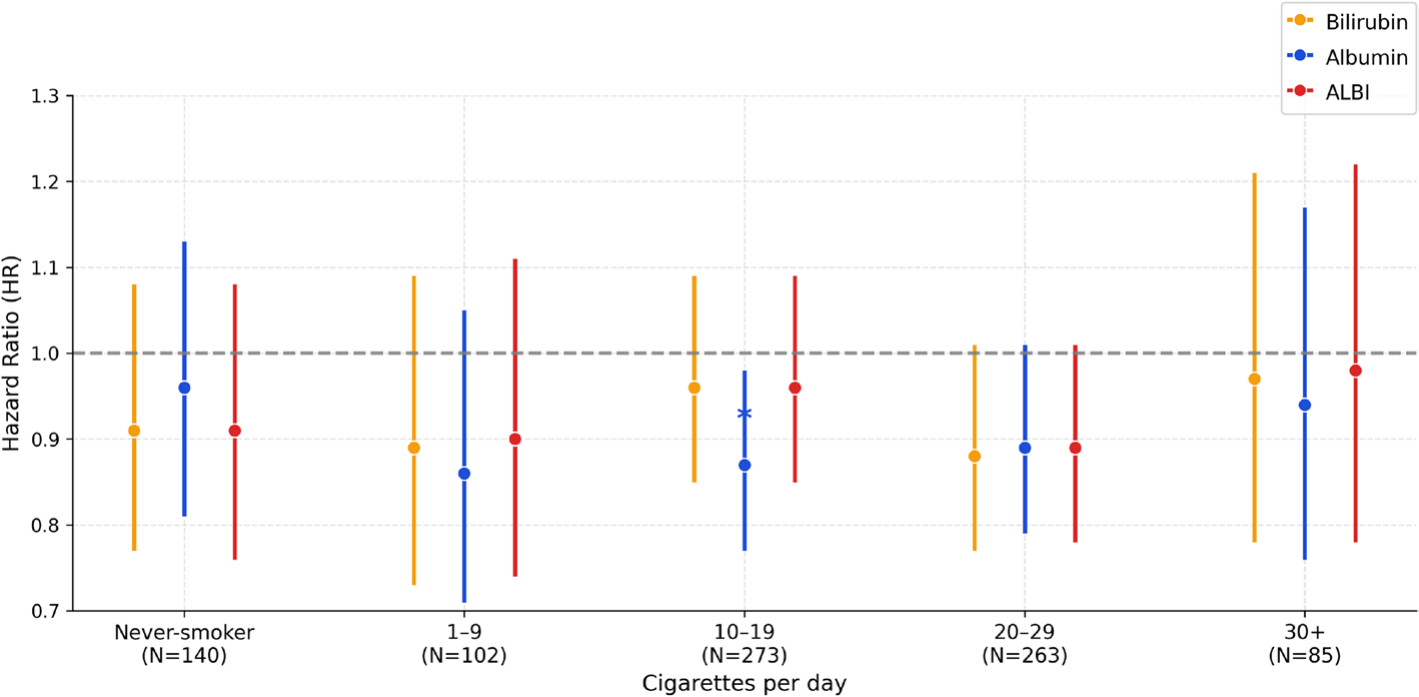
Effect of total bilirubin, albumin, and ALBI on gastric cancer risk by smoking intensity in men: results from interaction analyses

In the subgroup analysis by smoking intensity, albumin showed a statistically significant inverse association among those who smoked 10–19 cigarettes per day (HR: 0.87, 95% CI: 0.77–0.98). In heavy smokers (20–29 cigarettes/day), all biomarkers showed borderline significant inverse associations (Fig. 2).

In the smoking-adjusted analysis of all men and women, albumin showed statistically significant inverse associations in both men (HR: 0.63, 95% CI: 0.49–0.81, *p* = 0.0002) and women (HR: 0.60, 95% CI: 0.37–1.00, *p* = 0.0476) (Supplementary Fig. 2).

In summary, inverse associations between serum antioxidant biomarkers and gastric cancer risk were more prominent in smoking-exposed groups, with albumin consistently showing the strongest and most statistically significant associations.

## Discussion

This study is the first prospective cohort investigation to evaluate the associations of total bilirubin, albumin, and the albumin-bilirubin (ALBI) index with gastric cancer risk in a healthy male population, stratified by smoking status. Gastric cancer is one of the most common and deadly malignancies worldwide, and its development is known to be influenced by a variety of environmental and biological factors [1, 29]. Among these, smoking is a well-established risk factor. Numerous epidemiological studies and meta-analyses have consistently demonstrated a significant positive association between smoking intensity or duration and gastric cancer risk [3, 5, 6]. In our study, the interaction between smoking quantity and gastric cancer risk was also observed in men, with a statistically significant association particularly evident among those who smoked 10–29 cigarettes per day (Fig. 2).

Total bilirubin, albumin, and the ALBI index were significantly and inversely associated with gastric cancer risk among ever-smokers (including both current and former smokers). These inverse associations were consistent not only in the analyses per 1-standard deviation (SD) increase, but also in quartile-based and trend analyses (p for trend). Albumin showed the strongest inverse association, which remained robust in sensitivity analyses adjusted for socioeconomic factors such as income and education level among male smokers. The association also persisted in the overall population of men and women after adjusting for smoking status (Supplementary Fig. 2). In contrast, no significant associations between these biomarkers and gastric cancer risk were observed among never-smokers. This finding suggests that the pathophysiology of gastric cancer may differ by smoking status. However, considering the relatively small sample size and low incidence of gastric cancer in the never-smoker group, the absence of significant associations in this subgroup may be partially attributed to limited statistical power. In smokers, the accumulation of reactive oxygen species (ROS) and the resulting oxidative stress may play a central role in gastric carcinogenesis, whereas in never-smokers, other mechanisms such as Helicobacter pylori infection, dietary factors, genetic susceptibility, and chronic inflammation may be more influential [1, 6, 11–14, 30]. Moreover, gastric cancer is a heterogeneous disease, with transcriptomic as well as molecular and clinical characteristics varying not only by anatomical location (cardia vs. non-cardia) and histological type (intestinal vs. diffuse) but also across different stages of disease progression. In light of this, future studies should incorporate progression-dependent analyses to more precisely elucidate the relationships between biomarkers and gastric cancer risk [31].

Bilirubin is a key endogenous antioxidant molecule that helps maintain NADH/NAD⁺ redox balance, scavenges ROS, inhibits protein phosphorylation, stabilizes cell membranes, and protects mitochondria, thereby reducing cellular damage and preventing the formation of a tumor-promoting microenvironment [32–40]. Notably, Mölzer et al. reported that bilirubin exhibits antimutagenic effects in gastrointestinal tissues, supporting its biological protective role in gastric cancer development [41]. Furthermore, bilirubin may exert long-term protective effects by modulating chronic oxidative and inflammatory stress associated with gastric tumorigenesis [40]. Albumin also acts as a potent antioxidant protein by binding metal ions, thereby inhibiting ROS generation via the Fenton reaction and neutralizing free radicals [42]. In chronic smokers who are continuously exposed to oxidative and inflammatory stress, the antioxidant capacity of albumin may play an especially important physiological role. The strong inverse association observed between albumin and gastric cancer risk in our study suggests that albumin may function as more than just a nutritional marker and could serve as a key component of the body’s antioxidant defense system. Additionally, bilirubin and albumin may exert a synergistic effect by inhibiting activation of the NF-κB signaling pathway and suppressing the release of pro-inflammatory cytokines such as TNF-α and IL-6, thereby mitigating the formation of a pro-tumor inflammatory microenvironment [43]. The ALBI index was originally developed to assess liver function and predict prognosis in patients with hepatocellular carcinoma, based on a simple formula combining total bilirubin and albumin levels [20]. In the present study, we interpreted the ALBI index as a composite biomarker reflecting the antioxidant properties of its two components and applied it, for the first time, to a healthy general population. By evaluating ALBI levels prior to cancer onset, our study offers a novel perspective distinct from previous prognosis-oriented research, highlighting the potential preventive implications of ALBI as an antioxidant marker.

In summary, this study demonstrates that total bilirubin, albumin, and the ALBI index are significantly and inversely associated with gastric cancer risk among smokers, suggesting their potential involvement in the carcinogenic process under oxidative stress conditions. Future studies are needed to assess whether these findings are generalizable to women, individuals of different ethnic backgrounds, pre- and postmenopausal populations, and those with diverse genetic susceptibilities. Furthermore, mechanistic investigations are warranted to elucidate the biological pathways through which these biomarkers may contribute to gastric cancer development.

### Limitations

This study has several limitations. First, serum bilirubin and albumin levels were measured at a single time point, which may not fully reflect long-term exposure or temporal changes in antioxidant status. Second, bilirubin levels can be influenced by various factors such as liver function, hemolysis, and genetic polymorphisms, including UGT1A1 variants associated with Gilbert’s syndrome, which were not fully accounted for in this study. Third, several established gastric cancer risk factors were not included in the analysis, including *Helicobacter pylori* infection, dietary factors (such as salt intake and processed meat consumption), vitamin C status, gastrointestinal disease history, psychological stress, inflammatory conditions, and medication use, which may result in residual confounding. Fourth, although smoking exposure was quantified using smoking duration, smoking intensity, and pack-years among ever smokers, detailed information on smoking cessation and temporal changes in smoking behavior was not available. Fifth, the study population consisted of middle-aged Korean men who underwent health screenings, which may limit the generalizability of the findings to women or other populations with different genetic backgrounds, dietary patterns, and lifestyle factors. Finally, the ALBI index was originally developed to assess liver function and prognosis in hepatocellular carcinoma patients. In this study, it was interpreted as a biomarker reflecting antioxidant potential in relation to gastric cancer risk, and therefore its application as a predictive marker for gastric cancer should be interpreted with caution.

## Conclusions

This study reinterpreted total bilirubin, albumin, and the ALBI index as antioxidant-related biomarkers and evaluated their associations with gastric cancer risk according to smoking status. Among male smokers, all three biomarkers showed significant inverse associations with gastric cancer risk, with albumin demonstrating the strongest protective effect. In contrast, no significant associations were observed among never-smokers. These findings provide a foundation for the clinical interpretation of antioxidant biomarkers in populations exposed to chronic oxidative stress and may offer valuable evidence for early identification and risk stratification of individuals at high risk for gastric cancer. Future studies are warranted to validate these findings in diverse populations and to elucidate the underlying biological mechanisms through which antioxidant biomarkers influence gastric carcinogenesis. In particular, longitudinal studies incorporating additional risk factors such as Helicobacter pylori infection and dietary exposures are needed to further clarify these associations.

## Supplementary Information

Additional file 1: Supplementary Figure 1. Flow diagram of study participants. Supplementary Figure 2. Associations of Total Bilirubin, Albumin, and ALBI with Gastirc Cancer Risk in Men and Women.

Additional file 2: Supplementary Table 1. Associations of Antioxidant Biomarkers with Gastric Cancer Risk by Smoking Status in Men: Sensitivity Analysis with Socioeconomic Adjustment.

## Data Availability

The datasets, including summary statistics and R code generated during the current study, are available from the corresponding author upon reasonable request. Due to privacy and ethical restrictions, raw data are not publicly available.

## Abbreviations

ALBI: Albumin–bilirubin (index)
BMI: Body mass index
CI: Confidence interval
CV: Coefficient of variation
DPD: 3,5-Dichlorophenyldiazonium tetrafluoroborate
GGT: Gamma-glutamyl transferase
GOT: Glutamate oxaloacetate transaminase
HR: Hazard ratio
ICD-10: International Classification of Diseases, 10th Revision
IQR: Interquartile range
KCPS-II: Korean Cancer Prevention Study-II
NADH/NAD⁺: Reduced nicotinamide adenine dinucleotide / nicotinamide adenine dinucleotide (oxidized form)
NCC: National Cancer Center
NF-κB: Nuclear factor kappa B
ROS: Reactive oxygen species
SD: Standard deviation
TNF-α: Tumor necrosis factor alpha
UGT1A1: UDP-glucuronosyltransferase family 1 member A1
WHO: World Health Organization
