# Supplementary material for: Associations of bilirubin, albumin, and the ALBI index with gastric cancer risk: a smoking-stratified prospective cohort study in men
Source: BMC Gastroenterol. 2026 Jun 11;26:510. doi: 10.1186/s12876-026-05002-1 (PMC13479926; doi:10.1186/s12876-026-05002-1)
Supplement: Supplementary file 1 [file 12876_2026_5002_MOESM2_ESM.docx]

Supplementary Table 1. Associations of Antioxidant Biomarkers with Gastric Cancer Risk by Smoking Status in Men: Sensitivity Analysis with Socioeconomic Adjustment.

| Biomarkers | Smoking  Status | Case | HR (95% CI) | *p*-value |
| --- | --- | --- | --- | --- |
| Bilirubin | †Never-Smokers | 49 | 1.19 (0.60-2.35) | 0.6137 |
|  | †Former Smokers | 167 | 0.72 (0.46-1.13) | 0.1521 |
|  | †Current Smokers | 178 | 1.09 (0.73-1.64) | 0.6773 |
|  | †Ever-Smokers* | 345 | 0.83 (0.62-1.12) | 0.2259 |
|  | ‡All men | 394 | 0.92 (0.71-1.21) | 0.5669 |
| Albumin | †Never-Smokers | 49 | 1.03 (0.36-2.99) | 0.9552 |
|  | †Former Smokers | 167 | 0.71 (0.41-1.23) | 0.2191 |
|  | †Current Smokers | 178 | 0.41 (0.24-0.69) | 0.0008 |
|  | †Ever-Smokers* | 345 | 0.51 (0.35-0.75) | 0.0006 |
|  | ‡All men | 394 | 0.58 (0.40-0.83) | 0.0027 |
| ALBI | †Never-Smokers | 49 | 1.19 (0.36-3.92) | 0.7785 |
|  | †Former Smokers | 167 | 0.72 (0.37-1.37) | 0.3150 |
|  | †Current Smokers | 178 | 0.92 (0.50-1.69) | 0.7762 |
|  | †Ever-Smokers* | 345 | 0.74 (0.48-1.14) | 0.1734 |
|  | ‡All men | 394 | 0.85 (0.56-1.30) | 0.4581 |

Abbreviations: CI, confidence interval; HR, hazard ratio.

*Ever-smokers include both current and former smokers.

†The Cox proportional hazards model was adjusted for age, alcohol drinking, body mass index, GOT, GGT, family history of cancer, income and education.

‡The Cox proportional hazards model was adjusted for age, smoking status, alcohol drinking, body mass index, GOT, GGT, family history of cancer, income and education.
